# Two-dimensional nanoframes with dual rims

**DOI:** 10.1038/s41467-019-13738-6

**Published:** 2019-12-19

**Authors:** Sungjae Yoo, Jeongwon Kim, Sungwoo Choi, Doojae Park, Sungho Park

**Affiliations:** 1 Department of Chemistry, Sungkyunkwan University, Suwon, 440-746 South Korea; 2 Department of Applied Optics and Physics, Hallym University, Chuncheon, 24252 South Korea

**Keywords:** Nanoparticles, Synthesis and processing

## Abstract

The synthesis of highly complex two-dimensional (2D) metal nanoframes remains a great challenge. Synthetic strategies for preparing 2D metal nanoframes are few, and rational and systematic synthetic pathways to more complicated architectures have not yet been reported. Herein, we demonstrate a stepwise synthetic strategy for complex 2D metal nanoframes with a high degree of intricacy; the strategy leads to a variety of shapes, including rings, triangles, hexagons, and tripods with tailorable single or double frames in a single entity. These nanoframes of high homogeneity could be obtained through selective combination of four different chemical toolkits consisting of selective etching and deposition on certain facets, and concentric and/or eccentric regrowth by controlling the mismatches of lattice constants of metals. The resulting nanoframes were highly homogeneous in size and shape and had van der Waals interactions that maximized rim-to-rim contact, allowing them to uniquely self-assemble into large-area superstructures.

## Abstract (web summary)

The synthesis of anisotropic nanoparticles with structural diversity and complexity in a rational and systematic fashion is rare. Here, the authors demonstrate a rational and stepwise synthetic strategy for nanoplates with a high degree of intricacy, leading to a gallery of shapes such as rings, triangles, hexagons, and tripods, with tailorable single or double frames.

## Introduction

Similar to the fact that all planets are spherical, owing to the gravity well centered on the body’s center of mass, in most cases the shapes of metallic nanoparticles are likewise limited to spherical geometry owing to self-stabilization that minimizes their exposed surface area. The synthesis of anisotropic nanoparticles that deviate from this spherical shape, and especially nanoplate-like architectures^1–5^, remains a great challenge. Further, producing nanoplates with structural diversity and complexity in a rational and systematic fashion is very rare. To synthesize sophisticated architectures of high-order complexity and homogeneity^6–10^, tour de force synthetic methods are required. Multiple-step reactions are the typical strategy used to synthesize such complex nanostructures, although the examples referenced above often adopt repeated instances of the same synthetic steps, resulting in limited success in forming complex nanoparticles. Sequential reactions composed of multiple distinct synthetic pathways^11^ and the ability to combine such steps on demand will offer a powerful tool for further expanding the variety of 2D nanoplate architectures with a high degree of intricate features, mirroring the synthetic strategies often adopted in the total synthesis of complex organic molecules. Herein, we demonstrate a rational and stepwise synthetic strategy for nanoplates with an high degree of intricacy, leading to a gallery of various shapes such as rings, triangles, hexagons, and tripods, and having tailorable single or double frames. These high homogeneous nanoframes can be obtained through selective combinations of four different chemical toolkits consisting of selective etching, rim-on deposition, and concentric and/or eccentric regrowth.

## Result and discussion

### Synthetic strategy of 2D complex nanoframes

In the selective etching step, one component could be etched out leaving the other component almost intact, which is useful for obtaining frameworks. In addition, the selective etching could be performed on the reshaping process of the synthesized nanoparticles. For instance, the tips or protruded domains of nanoparticles could be selectively etched out, leading to the rounded-shaped of nanoparticles, which opens up the way of reshaping the nanoparticles. In the step of rim-on deposition, one can control the way of deposition of another component on the certain facets of template nanoparticles taking advantage of surface energy and/or coordination number difference. Typically, Pt^4+^ ions are more likely reduced on the facets with the lower coordination number like the edge and vertex, as compared to the terrace of Au nanoparticles. Another important synthetic strategy is the control of regrowth direction. In our 2D nanoframes, the complexity of nanoframes could be further evolved by controlling the regrowth pattern of Au around Pt skeleton. When AuCl_4_^−^ ions are added to reaction solution containing the two-component PtAu nanoparticles in the presence of reductants, the AuCl_4_^−^ ions are selectively reduced on Au domains, due to the large lattice constant mismatch between Au and Pt (*vide infra*). However, the homogeneous deposition of Au on PtAu nanoparticles is also feasible by depositing a thin layer of Ag (as a mediator reducing the lattice mismatch between Pt and Au) on Pt. By adopting this synthetic strategy, one can control the Au regrowth direction around PtAu nanoframes, saying eccentric or concentric growth (*vide infra*). Importantly, one can combine these four synthetic steps and perform them multiple times to control the complexity of nanoparticles as following; (1) Selective-etching: one metal is selectively etched to ions, leaving the other metal almost intact. (2) Rim-on-deposition: metal ions are exclusively reduced at the periphery of the core plate. (3) Concentric-growth: metal atoms are homogeneously deposited around the metal frames. (4) Eccentric-growth: metal atoms are selectively deposited to certain domains of metal frames wherein the difference of lattice constants of metals is absent.

### Fine-control of 2D Au nanoplate morphology

**Fig. 1:**
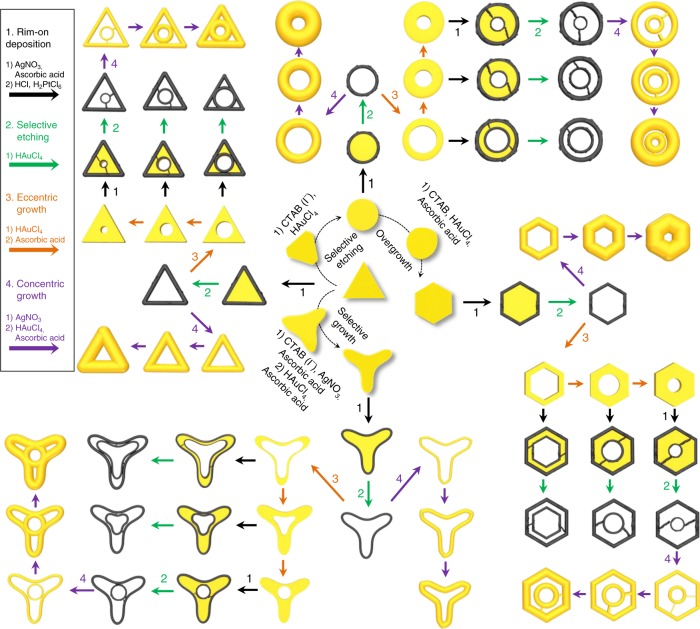
Schematic illustration of the synthetic pathways of complex 2D nanostructures. The multistep reactions to synthesize complex 2D metal nanoframes involved rim-on deposition of Pt (black arrow), selective etching (green arrow), eccentric growth (orange arrow), and concentric growth (purple arrow).

Figure 1 shows the complete chemical synthetic pathways for complex 2D metal nanoframes, starting from triangular Au nanoplates and going through multiple synthetic steps on demand, systematically leading to complex 2D nanostructures with double nanoframes. Au nanoplates of various shapes (triangle, disk, hexagon, and tripod), as represented in the scheme, were adopted as starting templates en route to 2D complex nanoframes. Fine-tuning in the resulting shapes could be achieved by controlling the etching and growth parameters precisely (*vide infra*). We designate the corresponding shape-adjustment steps as selective etching, selective growth, and overgrowth modes, as described in the central region of Fig. 1. In the selective etching step, AuCl_4_^–^ ions were added to the triangular nanoplates solution; the Au nanoplates initially showed sharp three vertices and gradually became truncated (AuCl_4_^−^ ^+^ 2Au + 2Cl^−^ → 3AuCl_2_^−^), eventually leading to disks. It also implies that the standard redox potential of the oxidation of vertex is lower than that of AuCl_4_^−^ and therefore the oxidation of vertex (Au_vertex_(s) +s2Cl^−^ ^→^ AuCl_2_^−^ uC2e^−^) occurs as AuCl_4_^−^ ions become reduced^12^. In the overgrowth step, when the chemical environment was changed to mild reduction conditions by slowly adding ascorbic acid, Au atoms preferred to be deposited on the edges of disks (which had higher surface energy compared to flat terraces) and the shape evolved to a hexagonal shape. Alternatively, in the selective growth step, a thin layer of Ag was formed on the {111} facets (top and bottom terraces) of triangular Au nanoplates in the presence of I^–^ ions, and then ascorbic acid (reducing agent) and AuCl_4_^–^ ions were added sequentially. It is noteworthy that AuCl_4_^–^ ions are reduced to AuCl_2_^–^ ions in the presence of ascorbic acid. The existence of a thin Ag layer on Au nanoplates determined the growth mode of Au, exclusively on the tips, preventing the Au growth on terraces., whereby Ag^+^ ions were reduced again to a thin Ag layer by ascorbic acid. At the same time, the edge sites of Au nanoplates were oxidized by residual AuCl_4_^–^ ions (still unreacted with ascorbic acid), leading to partial etching of edges, and thus eventually to tripods^13,14^. It is noteworthy that the observed shape transformation of triangular Au nanoplates proceeded without any new nucleation events in all synthetic processes. The average size of the newly formed nanoplates depends on the amount of etchants (AuCl_4_^–^ for disks) and/or precursors (AuCl_2_^–^ for hexagons and tripods) present in the solution. Typically, the diameter (distance from the center to the tip) can be tuned within the range from 78 ± 5 nm to 54 ± 5 nm and the thickness is from 18 ± 1 nm to 10 ± 2 nm (Supplementary Fig. 1).

### Synthetic steps in the synthesis of 2D complex nanoframes

In Fig. 1, each distinctive step in the synthesis of complex nanoframes is described by arrows having different colors for easier recognition; these are listed as follows. (1) Rim-on deposition (black arrow): metal ions are exclusively reduced at the periphery of the core plate. (2) Selective etching (green arrow): one metal is selectively etched to ions, leaving the other metal almost intact. (3) Eccentric growth (orange arrow): metal atoms are selectively deposited to certain domains of metal frames where there is no difference in the lattice constants of metals. (4) Concentric growth (purple arrow): metal atoms are homogeneously deposited around the metal frames. By following the schematic pathways described in Fig. 1, we will describe how one can synthesize the complex 2D nanoframes with dual rims in a single entity, composed of six steps as follows.

### A section for rim-on deposition

In step one (rim-on deposition), when Pt^4+^ ions were added to Au nanoplates coated with thin layer of Ag (triangle, disk, hexagon, or tripod shapes) in the presence of ascorbic acid, the galvanic replacement reaction between Pt^4+^ ions and the thin layer of Ag on Au surface occurred exclusively on the vertices and edges due to the higher surface energy of {112} facets (i.e., the vertices and edge) compared to {111} facets (i.e., the terraces on top and bottom) of nanoplates (Supplementary Fig. 2)^15^. The thickness of Pt rims at the peripheries could be tailored by the total amount of Pt^4+^ ions added to the reaction solution. Typically, we controlled the Pt rim thickness to be around 11 ± 2 nm (disk), 12 ± 3 nm (triangle), 11 ± 2 nm (hexagon), and 10 ± 4 nm (tripod) (Supplementary Fig. 3).

### A section for selective etching

**Fig. 2:**
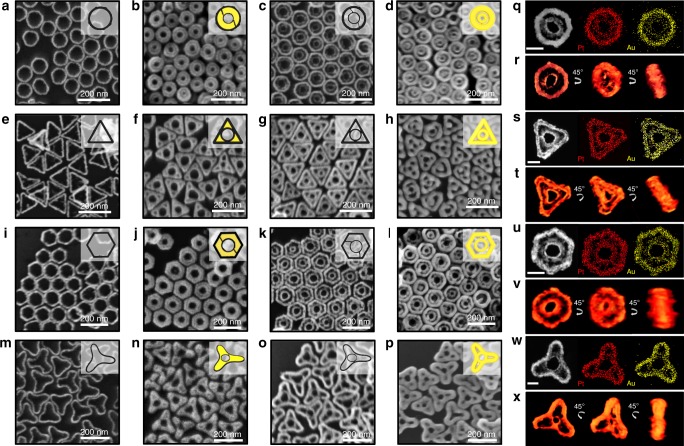
Morphology evolution of 2D nanoframes. FE-SEM images of (**a**, **e**, **i**, and **m**) 2D PtAu single nanoframes, (**b**, **f**, **j**, and **n**) 2D Pt@Au@Pt nanoframes, (**c**, **g**, **k**, and **o**) 2D PtAu double nanoframes, and (**d**, **h**, **l**, and **p**) 2D Pt@Au double nanoframes with different shapes. (**q**, **s**, **u**, and **w**) TEM images and EDS image mappings of 2D PtAu double nanoframe with different shapes (scale bars: 50 nm). (**r**, **t**, **v**, and **x**) 3D visualizations of 2D PtAu double nanoframes from the STEM tomography.

Next, in step two (selective etching), the central bulk Au domains were selectively etched to Au^+^ by means of adding AuCl_4_^–^ ions (AuCl_4_^–^ + 2Au^0^ + 2Cl^–^ ↔ 3AuCl_2_^–^), while the Au adatoms in close proximity to Pt rims remained intact, leaving a PtAu framework (Fig. 2a, e, i, and m). The incomplete etching of inner Au domains to a very thin layer was readily achievable and controllable, possibly because the Au adatoms at the boundary between the Pt rim and Au domain intercalate to form an alloy between Pt and Au at the boundary, leaving a very thin layer of Au in the inner rims. Energy-dispersive spectrometry (EDS) mapping images revealed that there was ca. 73.95% and 26.05% (disk), 52.61% and 47.39% (triangle), 58.96% and 41.04% (hexagon), and 45.96% and 55.04% (tripod) Pt and Au atoms, respectively, in the single nanoframes (Supplementary Fig. 3). As in the case of single nanoframes, the complete removal of Au was not feasible due to PtAu alloy formation. Although elemental mapping showed Au on the Pt rims, the pure Au was too thin to show the corresponding optical characteristics of Au, as evident from the surface plasmon profile of the single nanoframes acquired by means of UV/Vis-NIR spectroscopy (Supplementary Fig. 4). When the Au atoms were deposited on the rims of 2D PtAu single nanoframes, there were two alternative growth patterns (i.e., concentric or eccentric growth modes). In the eccentric growth mode, Au atoms were preferentially deposited on the inner regions of the PtAu nanoframes. In contrast, in the concentric growth mode, Au atoms were deposited almost equally on all directions. These two different growth patterns could be directed by controlling the reaction parameter (*vide infra*).

### A section for eccentric growth

**Fig. 3:**
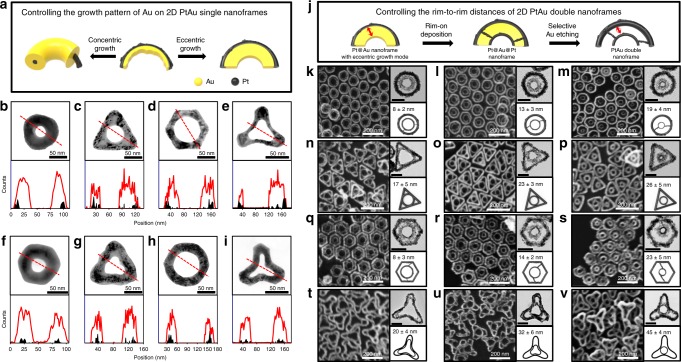
Controlling the growth pattern of Au and rim-to-rim distances of PtAu double nanoframes. **a** Schematic illustration shows the mechanism of the growth pattern control of Au on 2D PtAu single nanoframes. TEM images and line mapping profiles show 2D Pt@Au nanoframes grown in (**b**–**e**) eccentric and (**f**–**i**) concentric growth modes. Line mapping was conducted along the red lines in TEM images. **j** Schematic illustration of shows the synthesis procedure of 2D PtAu double nanoframes with various shape and rim-to-rim distances. **k**–**v** FE-SEM images and TEM images show 2D PtAu double nanoframes having various rim-to-rim distances in each shape. Morphology and dimension information (rim to rim distances) are given below each TEM image (scale bars: 50 nm).

In step three (eccentric growth), when AuCl_4_^–^ ions were added to PtAu nanoframes, the residue of Au on the inner rims acted as a nucleus and the AuCl_4_^–^ ions were selectively reduced on Au domains, resulting in homogeneous inward Au growth (Fig. 3b–e). As Au growth proceeded, there was no noticeable change in the outer diameter (from 97 ± 6 nm to 100 ± 7 nm); rather, the inner diameter of the empty holes decreased while retaining its circular shape, indicating that the growth mode followed the Frank–van der Merwe mode, wherein adatoms attach preferentially to surface sites to produce a smooth and fully formed layer (Supplementary Fig. 5). It is noteworthy that the growth of Au in the outward direction was not favorable because the lattice constant mismatch between Au and Pt is relatively large (i.e., the lattice constant of Au and Pt is 0.4065 nm and 0.3912 nm, respectively). EDS line mapping (Fig. 3, below each TEM image) showed that, regardless of shape, the Pt domains were dominantly located on the outer sides of rims, whereas Au resided on the inner sides of rims. Importantly, the inner Au domain thickness could be controlled precisely by controlling the total amount of Au^3+^ ions, allowing one to control the diameter of inner empty holes. Typically, for rings, the thickness of Au domains was controlled from 19 ± 3 nm to 39 ± 3 nm (Supplementary Fig. 5).

In step four (rim-on deposition), again, when Pt^4+^ ions were added to Au rings (triangle, disk, hexagon, or tripod shapes) in the presence of ascorbic acid, Pt atoms were deposited on both the inner and outer peripheries, excluding deposition on the flat tops and bottoms of Au domains. The resulting Pt@Au@Pt single nanoframes are shown in Fig. 2b, f, j, and n(second column).

In step five (selective etching), the inner Au domains were selectively etched to Au^+^ by the addition of AuCl_4_^–^ ions, leaving a PtAu framework with double rims in one entity, as shown in Fig. 2c, g, k, and o (third column). It is remarkable that the inner rims were interconnected with the outer rims through the scattered PtAu islands without the loss of integrity of the double frame architecture (for ring and hexagon shapes). For triangles and tripods, triangle-inscribed and tripod-inscribed circles were clearly observed to be in contact with the three corresponding edges. The intricacy of double frames could be evolved further by means of controlling the thickness of inner Au domains as described in Fig. 3j^16–19^ Strikingly, the inner rim–to–outer rim distance could be tailored by controlling the widths of the inner Au domains. Typically, the rim-to-rim distance for a disk shape was tunable from 8 ± 2 nm to 19 ± 4 nm (Fig. 3k–m). For the triangle shape, the three holes at the tips were distinctive when the tip-to-inner rim distance was 17 ± 5 nm, and they gradually merged as the tip-to-inner rim distance approached 26 ± 5 nm (Fig. 3n–p). This tunability was applicable to hexagons and tripods as well (Fig. 3q–v). Low-magnification field-emission scanning electron microscopy (FESEM) images revealed that the as-prepared double nanoframes were extraordinarily homogeneous in both shape and size, indicating the efficacy and controllability of the suggested multistep synthetic routes (Supplementary Fig. 6). The morphologies of PtAu double nanoframes were further analyzed in detail from different angles using angle-dependent scanning transmission electron microscopy (STEM) tomography (Fig. 2r, t, v, x and Supplementary Movies 1–4), and their two-dimensional geometry and compositional information were determined (Supplementary Fig. 7).

### A section for concentric growth

In step six (concentric growth), when AuCl_4_^–^ ions were added to Pt nanoframes in the presence of ascorbic acid, the AuCl_4_^–^ ions were homogeneously deposited around the Pt rims, resulting in Au growth both inward and outward. The Ag present on the Pt rims played the role of a mediator, reducing the difference in lattice constants between Pt and Au and thereby allowing the deposition of Au not only on Au but also on Pt to an equal extent (note that the lattice constant of Ag is 0.4079 nm). We added Ag ions and Au ions to the PtAu nanoframe solution at the same time and then added ascorbic acid. In this step, an Ag UPD layer was formed on the entire surface of PtAu nanoframes, and then Au ions were subsequently deposited on the whole surface of PtAu nanoframe^20,21^. No galvanic replacement reaction was observed. We believe that when the thick layer of Ag exists, the galvanic replacement is favorable with AuCl_4_^+^ ions added. However, the very thin layer of Ag behaves differently as compared to the thinker Ag layer under the same experimental conditions. The resulting Au double nanoframes (Fig. 2d, h, l, and p) exhibited thicker grown rims compared to Pt double frames (Fig. 2c, g, k, o and Supplementary Fig. 8). The successful concentric growth of Au was clearly evident in the corresponding UV-vis-NIR spectra, where the long-wavelength band was gradually hypochromically shifted and became more intense as the Au coating around the core Pt rims proceeded (Supplementary Figs. 9 and 10). When the concentric growth mode was applied to single nanoframes, the EDS line mapping showed Pt rims in the cores of rims (Fig. 3f–i), different from the case of eccentric growth mode. Interestingly, X-ray diffraction (XRD) patterns of Pt@Au single frames obtained by means of eccentric growth mode indicated only two different crystalline peaks (i.e., {111} and {222}), whereas Pt@Au rings synthesized by means of concentric growth exhibited several crystalline peaks (i.e., {111}, {200}, {220}, and {222}; Supplementary Fig. 11). The presence of a trace Ag layer facilitated faster deposition of Au on both Pt and Au domains, leading to the appearance of various crystalline facets.

### Electric-field simulation and assembly of 2D nanoframes

**Fig. 4:**
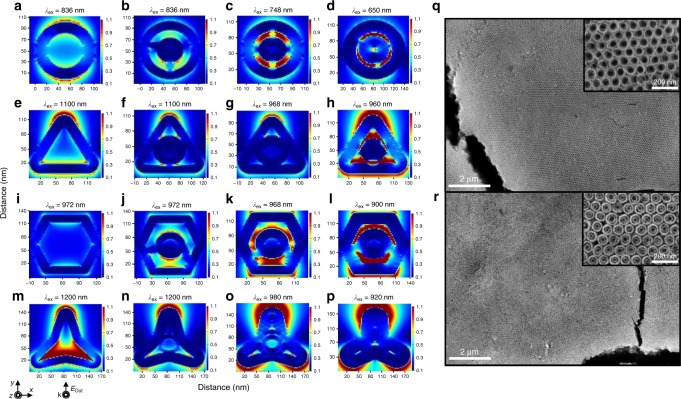
Electric field simulation and superstructures of 2D nanoframes. **a**–**p** Electric field enhancement contour maps around Pt@Au single and double nanoframes with different shapes and rim-to-rim distances; the incident wave vector k and the polarization vector E are indicated on the left bottom. FE-SEM images of the superstructure of (**q**) 2D PtAu single nanorings and (**r**) 2D PtAu double nanorings. Insets show zoomed-in images of the superstructures of 2D PtAu single nanorings and 2D PtAu double nanorings.

The Pt@Au double nanoframes were expected to show their unique near-field electromagnetic field coupling depending on the shape and the distance between the inner and outer rims (Fig. 4a–p). We carried out theoretical calculations using a conventional finite-difference time-domain method, finding that the electromagnetic field became coupled and enhanced as the rim-to-rim distance narrowed, regardless of shape. Interestingly, the field of rings with double frames was exclusively confined in the internal regions within nanoframes, whereas the triangular and hexagonal shapes exhibited tip-enhanced electromagnetic fields in addition to the internally confined field. In contrast, the tripod showed an internally confined field that gradually disappeared as the rim-to-rim gap narrowed. It is obvious that the capability of containing high volumes of molecules in the inner space between two rims and their unique electromagnetic fields inside and outside the cavity enable the design of highly effective surface-enhanced molecular sensing platforms^22–26^.

The resulting single or double nanoframes were exceptionally homogeneous in both size and shape, and therefore are expected to self-assemble into 3D-ordered superstructures^27–30^ during simple drop-casting and evaporation. Typically, the single and double nanoframes of ring shape exhibited phenomenal long-range order (of millimeter scale) and compactness with hexagonal pattern arrangement (exemplified by Fig. 4q, r, respectively). Careful investigation of their packing order revealed that the nanoframes were stacked in a rim-to-rim fashion, paving circular channels toward the bottom. It is well known that ordered pores have many useful applications such as in sorbents, supercapacitors, filters, and catalysts^31^.

In conclusion, we demonstrated a rational and stepwise synthetic strategy for complex, highly intricate nanoplate architectures, leading to rings, triangles, hexagons, and tripods with tailorable single or double frames, resembling total synthesis of complex organic molecules. The resulting 2D double nanoframes with large surface area and tunability in rim-to-rim distances are expected to open various applications in catalysis, nanophotonics, and biosensing^32–35^.

## Methods

### Synthesis of 2D Au triangular nanoplates

Au triangular nanoplates were prepared from 5 nm sphere seeds by means of a three-step seed-mediated method with iodide ions, as reported previously^2^. 500 μL of 20 mM aqueous HAuCl_4_·3H_2_O solution, 1 mL of a 10 mM aqueous solution of sodium citrate, and 1 mL of 100 mM aqueous NaBH_4_ (ice-cold) solution were added to 36.5 mL of deionized water under vigorous stirring. Three labeled flasks were used to prepare the triangular nanoplates. A mixture of 108 mL of 0.05 M aqueous CTAB solution and 54 μL of 0.1 M aqueous NaI solution was divided into three containers labeled 1, 2, and 3. 9 mL of the mixture were added each to containers 1 and 2, and the remaining 90 mL was added to container 3. A mixture of 125 μL of 20 mM aqueous HAuCl_4_·3H_2_O solution, 50 μL of 100 mM NaOH, and 50 μL of 100 mM ascorbic acid was then added each to containers 1 and 2. A mixture of 1.25 mL of 20 mM HAuCl_4_·3H_2_O, 0.5 mL of 100 mM NaOH, and 0.5 mL of 100 mM ascorbic acid was added to container 3. A total of 1 mL of the seed solution was added to container 1 under mild shaking, followed by the addition of 1 mL of the container 1 solution into container 2. After gentle shaking, all solution in container 2 was added to container 3.

### Synthesis of 2D Au circular nanoplates

Au circular nanodisks were prepared from triangular nanoplates by means of a comproportionation reaction. One and a half milliliters of a mixture of 10 mL of 0.1 M CTAB solution and 250 μL of 20 mM aqueous HAuCl_4_·3H_2_O solution was added to 10 mL of an aqueous solution of triangular nanoplates. The triangular nanoplates were etched via gold ions in an isothermal oven at 30 °C. After 1 h, the triangular nanoplates transformed to gold nanodisks. Residual gold ions were then removed by means of centrifugation (6511 × *g* for 20 min).

### Synthesis of 2D Au hexagonal nanoplates

Au hexagonal nanoplates were prepared from circular nanodisks by reducing HAuCl_4_ solution with ascorbic acid, as reported previously^36^. Au hexagonal nanoplates were obtained by mixing 1 mL of 0.1 M aqueous CTAB solution, 80 μL of 20 mM HAuCl_4_, 80 μL of 100 mM ascorbic acid and 6 mL of an aqueous solution of circular nanodisks. The major plasmon peak intensity of the aqueous solution of circular nanodisks was adjusted to 1.2 with water prior to mixing. The mixture was reacted by holding it in an isothermal oven at 30 °C for 10 h.

### Synthesis of 2D Au tripod nanoplates

Au tripod nanoplates were prepared from triangular nanoplates by means of site-selective regrowth with silver ions and etching of edge sites. 4 mL of an aqueous solution of triangular nanoplate, 20 mL of 0.05 M aqueous CTAB solution, 10 μL of 0.1 M aqueous NaI solution, 35 μL of 2 mM aqueous AgNO_3_, and 480 μL of 100 mM ascorbic acid were added to a 20 mL vial. The mixture was held in an isothermal oven at 70 °C for 1.5 h to deposit silver atoms onto the surfaces of the triangular nanoplates. After washing, the aqueous solution of Au@Ag triangular nanoplate was diluted to 15 mL by adding distilled water. Then, 2.5 mL of the aqueous solution of diluted Au@Ag triangular nanoplate, 5 mL of 0.05 M aqueous CTAB solution, 2.5 μL of 0.1 M aqueous NaI solution, 200 μL of 2 mM aqueous HAuCl_4_ solution and 100 μL of 100 mM ascorbic acid were added to a 20 mL vial. The resulting mixture was held in an isothermal oven at 70 °C for 3 h. The selective reduction step was monitored by means of extinction spectrum measurements. Residual ions were then removed by means of centrifugation (6511 × *g* for 20 min).

### Synthesis of 2D PtAu single nanoframes

The synthetic pathway for site-selective growth of Pt on Au nanoplates (Au@Pt nanoplates) followed our previously reported experimental procedure^15^. In the presence of iodide ions (50 μM), 20 mL of 0.05 M CTAB, 4 mL of redispersed Au nanoplates with different shapes (optical density of disk, prism, hexagonal, and tripod is 1.2, 1, 1, and 1, respectively), 20 μL (1.6 μM) of 2 mM aqueous AgNO_3_ solution, and 480 μL (2 mM) of 0.1 M aqueous ascorbic acid solution were added to a vial. The mixture was kept at 70 °C. After 1 h, 480 μL (2 mM) of 0.1 M HCl and 100 μL (8.3 μM) (disks), 175 μL (14.5 μM) (triangles), 150 μL (12.5 μM) (hexagons), and 250 μL (20.8 μM) (tripods) of 2 mM aqueous H_2_PtCl_6_ solution were added to the mixture with gentle shaking. The mixture was kept at 70 °C for 4 h. After this reaction, the sample was centrifuged and the supernatant was removed and redispersed in a mixture of 5 mL of 0.05 M CTAB aqueous solution and 2.5 μL of 0.1 M aqueous NaI solution. Next, 500 μL of 2 mM aqueous HAuCl_4_ was added to etch the Au@Pt nanoplates. This etching process was carried out for 1 h in a 50 °C oven and was followed by centrifugal washing (6511 × *g* for 20 min).

### Eccentric growth of 2D Pt@Au single nanoframes

Au frame nanoparticles with eccentric growth mode were prepared by reducing gold ions. A solution of Pt frame nanoparticles was stabilized by adding a mixture of 10 mL of 0.05 M CTAB aqueous solution and 5 μL of 0.1 M aqueous NaI solution. Aqueous 2 mM HAuCl_4_ solution was added to Pt frame nanoparticle solution in the following volumes: 200 μL (40 μM), 400 μL (80 μM), and 600 μL (60 μM) for disks. Then, 100 mM ascorbic acid was added to the resulting mixtures in a 1:10 volume ratio. The resulting reduction reaction was monitored by means of extinction spectrum measurements. Residual ions were then removed by means of centrifugation (6511 × *g* for 20 min).

### Synthesis of 2D PtAu double nanoframes

In the presence of iodide ions (50 μM), 20 mL of 0.05 M CTAB, 4 mL of redispersed inwardly grown Au frame nanoparticles of eccentric growth mode, 16 μL (1.3 μM) (disks), 15 μL (1.3 μM) (triangles), 16 μL (1.25 μM) (hexagons), and 12 μL (1 μM) (tripods) of 2 mM aqueous AgNO_3_ solution, and 480 μL (2 mM) of 0.1 M aqueous ascorbic acid solution were added to vials. Each resulting mixture was held at 70 °C. After 1 h, 480 μL (2 mM) of 0.1 M HCl and 96 μL (8 μM) (disks), 120 μL (10 μM) (triangles), 80 μL (6.7 μM) (hexagons), or 120 μL (10 μM) (tripods) of 2 mM aqueous H_2_PtCl_6_ solution were added to the respective mixtures with gentle shaking. Each sample was reacted at 70 °C for 4 h and then it was centrifuged two times. The supernatant holding the resulting Au@Pt nanoplates was removed and redispersed in a mixture of 4 mL of 0.05 M CTAB aqueous solution and 2.5 μL of 0.1 M aqueous NaI solution. Next, 250 μL (125 μM) of 2 mM aqueous HAuCl_4_ was added to etch the nanoplates. This etching process was carried out for 1 h in a 50 °C oven and was followed by centrifugal washing (6511 × *g* for 20 min).

### Growth of 2D Pt@Au double nanoframes

Pt@Au double nanoframes were prepared by means of Au regrowth on Pt frame nanoparticles with a small amount of silver ions. A solution of the synthesized PtAu frame nanoparticles was stabilized by adding a mixture of 4 mL of 0.05 M CTAB aqueous solution and 2.5 μL of 0.1 M aqueous NaI solution. Before the Au reduction reaction, 1 μL (0.5 μM) of 2 mM aqueous AgNO_3_ was added. Aqueous 2 mM HAuCl_4_ solution was added to Pt frame nanoparticle solution in the following volumes: 50 μL (25 μM), 100 μL (50 μM), and 150 μL (75 μM) for disks and prisms, 50 μL (25 μM), 100 μL (50 μM), and 200 μL (100 μM) for hexagons, and 60 μL (30 μM), 180 μL (90 μM), and 240 μL (120 μM) for tripods. Then, 100 mM ascorbic acid was added to the resulting mixtures in a 1:10 volume ratio. The resulting reduction reaction was monitored by means of extinction spectrum measurements. Residual ions were then removed by means of centrifugation (6511 × *g* for 20 min).

### Electric field simulations

Simulations of the electric field near the nanoframe were performed by means of a conventional FDTD method (FDTD Solutions, LumericalTM). Dimensions of nanoframes were indicated in Supplementary Fig. 10 for detail. The simulation volume of 140 nm × 140 nm × 1.3 μm was used to completely cover the nanoframe structure and the light source. A vertically polarized light source (polarized along the *y* direction in Fig. 4) of plane wave form for each simulated wavelength was positioned 300 nm away from the nanoframes, and a monitor of dimensions 123 nm × 123 nm was positioned at the center of the nanoframes. To ensure precise simulation, the minimum mesh size was set to 0.1 nm.

### Characterization

Field emission scanning electron microscopy (FESEM) images were obtained using JSM-7100F and JSM-7800F instruments (JEOL). JEM-2100F and JEM-ARM 200 F instruments (JEOL) were used to acquire transmission electron microscopy (TEM) images. UV-vis-NIR absorption spectra were acquired using a spectrophotometer (Shimadzu UV-3600). 3D tomography images were obtained using Talos F200X. A NX10 instrument (Park systems) was used to acquire AFM images and height profiles. X-ray diffraction (XRD) patterns of the nanostructures were characterized using a Rigaku Ultima IV. The electrochemical measurements were performed using an Auto Lab AUT12.

## Supplementary information

Supplementary Information

Description of Additional Supplementary Files

Supplementary Movie 1

Supplementary Movie 2

Supplementary Movie 3

Supplementary Movie 4

## Data Availability

The authors declare that all data are available from the corresponding authors on reasonable request
