# Supplementary material for: Two-dimensional nanoframes with dual rims
Source: Nat Commun. 2019 Dec 19;10:5789. doi: 10.1038/s41467-019-13738-6 (PMC6923375; doi:10.1038/s41467-019-13738-6)
Supplement: Supplementary file 1 — Supplementary Information [file 41467_2019_13738_MOESM1_ESM.pdf]

Supplementary Information

Two-Dimensional Nanoframes with Dual Rims

Yoo et al.

Table of Contents:

Supplementary Figures 1. FE-SEM images of prism nanoplates (A) and disk nanoplates (B)**…3**

Supplementary Figures 2. FE-SEM images of Au nanoplate and Au@Pt nanoplate of disk, triangle, hexagon, and tripod shapes**……………………..………………………..…………4**

Supplementary Figures 3. TEM images of 2D PtAu single nanoframes of disk, triangle, hexagon, and tripod shapes**………………………………………………….……………5**

Supplementary Figures 4. UV-vis-NIR spectra of Au nanoplates (red line), Au@Pt nanoplates (blue line), and single PtAu nanoframes (black line) of disk (A), triangle (B), hexagon (C) and tripod (D) shapes**………………………………………………………………………...6**

Supplementary Figures 5. FE-SEM images of PtAu single nanorings and Pt@Au single nanorings with different thickness of eccentric growth mode. And their corresponding UV-vis-NIR spectrum**…………………………………………………………………………...7**

Supplementary Figures 6. Low-magnification SEM images and size distributions of 2D PtAu double nanoframes of disk, triangle, hexagon, and tripod shapes**………………………..……..8**

Supplementary Figures 7. EDS data indicates elemental proportion of 2D PtAu double nanoframes of disk, triangle, hexagon, and tripod shapes**……………..……………..………9**

Supplementary Figures 8. TEM images and EDS mapping images of 2D Pt@Au double nanoframes of disk, triangle, hexagon and tripod shapes**…………..…..………..………..……10**

Supplementary Figures 9. FE-SEM images of PtAu double nanoframes and Pt@Au double nanoframes of disk, triangle, hexagon, and tripod shapes, which have concentric growth mode and their UV-vis-NIR spectra are obtained from the samples in SEM images**………………….11**

Supplementary Figures 10. Dimension of Pt@Au single nanoframes and Pt@Au double nanoframes with different shapes**…………………………………..……………………….12**

Supplementary Figures 11. Atomic force microscopy (AFM) images and X-ray diffraction (XRD) of Au nanorings with eccentric growth mode and concentric growth...**…..……………….13**

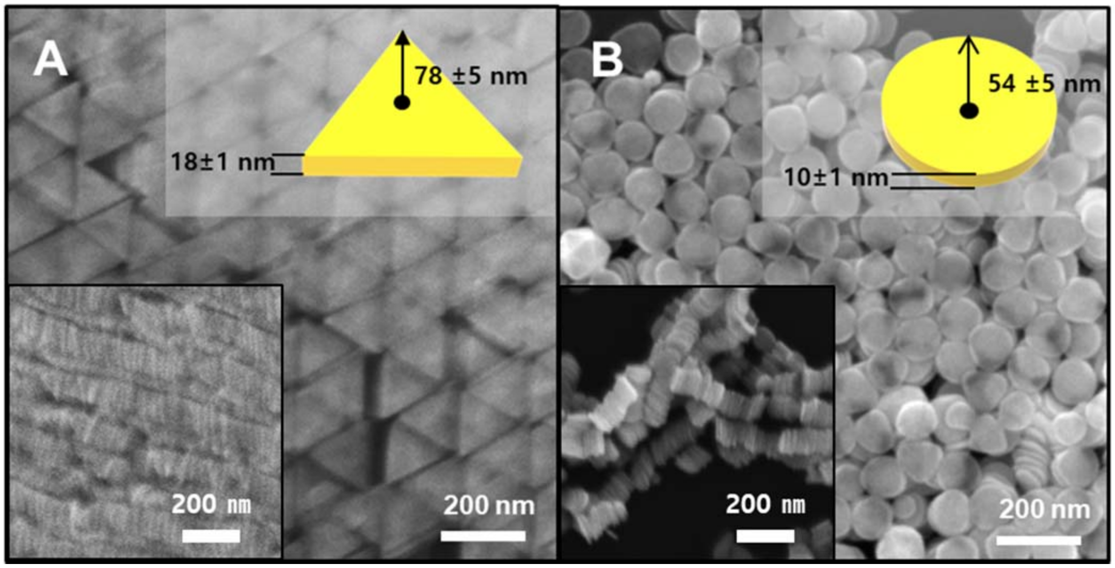

Supplementary Figures 1. FE-SEM images of prism nanoplates (A) and disk nanoplates (B). And each right upper inset shows the dimension (distance from center to tip and thickness is 78±5 nm and 18±1 nm, 54±5 nm and 10±1 nm, respectively) of nanoplates. Each left down inset shows side view of nanoplates

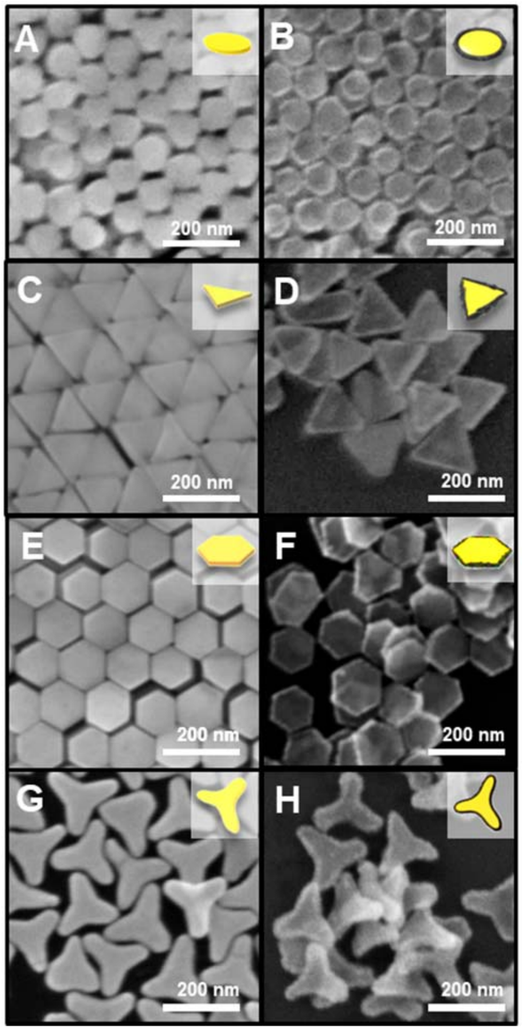

Supplementary Figures 2. FE-SEM images of Au nanoplate (A, C, E, and G) and Au@Pt nanoplate (B, D, F, and H) of disk, triangle, hexagon, and tripod shapes.

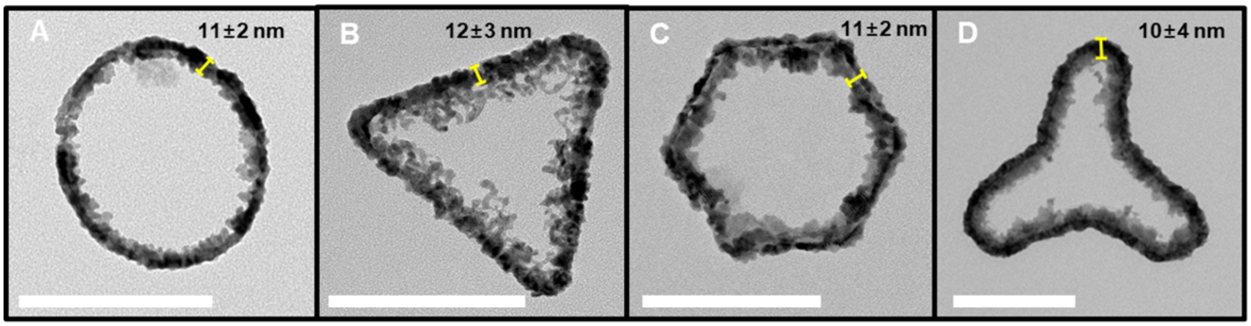

Element	Atomic %
Pt	73.95
Au	26.05
Total:	100.00

Element	Atomic %
Pt	52.61
Au	47.39
Total:	100.00

Element	Atomic %
Pt	58.96
Au	41.04
Total:	100.00

Element	Atomic %
Pt	45.96
Au	55.04
Total:	100.00

Supplementary Figures 3. TEM images of 2D PtAu single nanoframes of (A) disk, (B) triangle, (C) hexagon, and (D) tripod shapes. Scale bars: 100 nm.

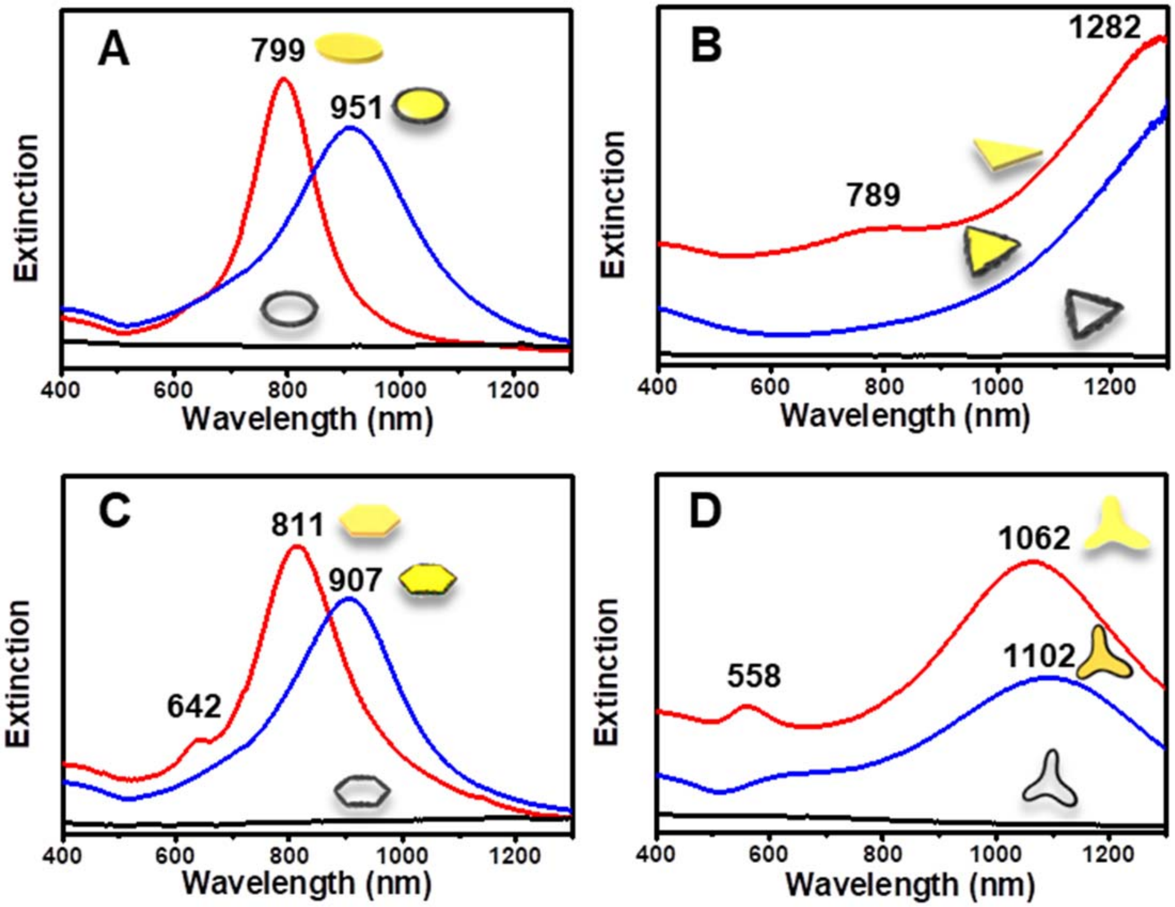

Supplementary Figures 4. UV-vis-NIR spectra of Au nanoplates (red line), Au@Pt nanoplates (blue line), and single PtAu nanoframes (black line) of disk (A), triangle (B), hexagon (C) and tripod (D) shapes.

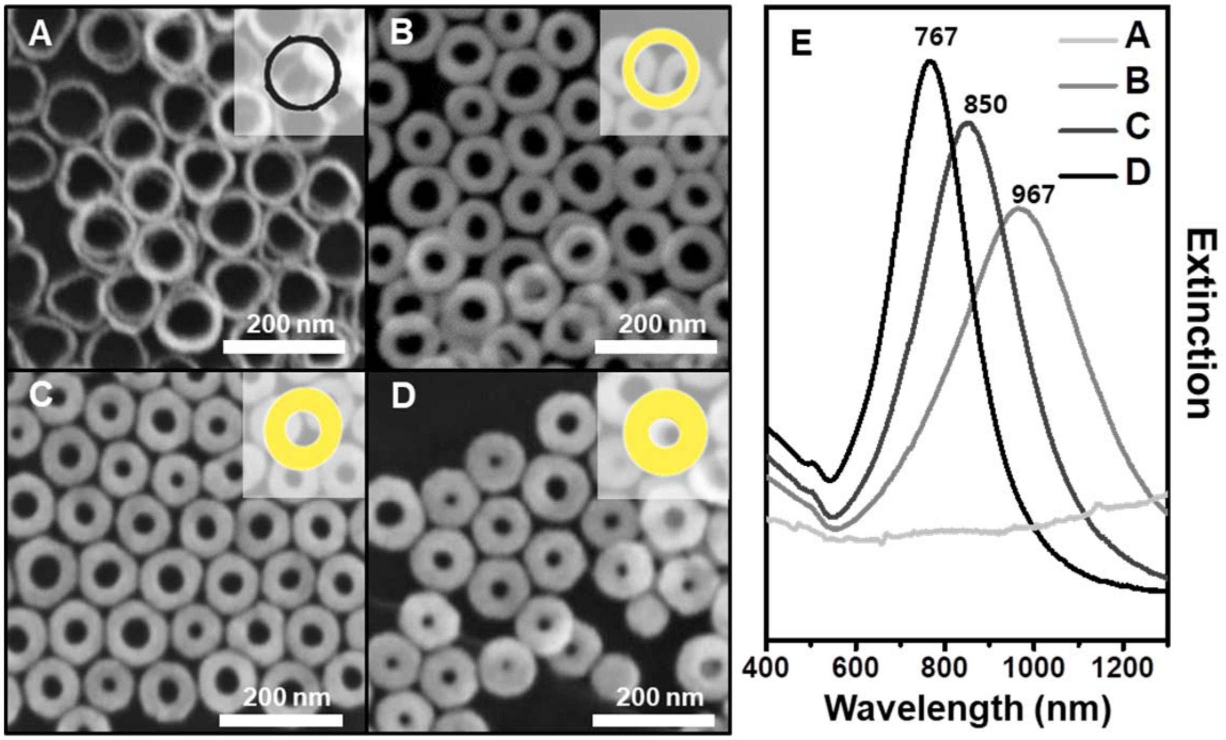

	A	B	C	D
Total diameter	97±6 nm	99±6 nm	99±6 nm	100±7 nm
Thickness of frame	19±3 nm	28±3 nm	32±4 nm	39±3 nm

Supplementary Figures 5. FE-SEM images of PtAu single nanrings (A) and Pt@Au single nanorings (B–D) of different thickness, depending on the concentration of $HAuCl_4$; 2 mM $HAuCl_4$ aqueous solution was added to the reaction solution in the volumes of (B–D) 200, 400 and 600 μL. (E) UV-Vis-NIR spectra obtained from the samples shown in the SEM images, in order of increasing Au^{3+} ions (from pale gray to black).

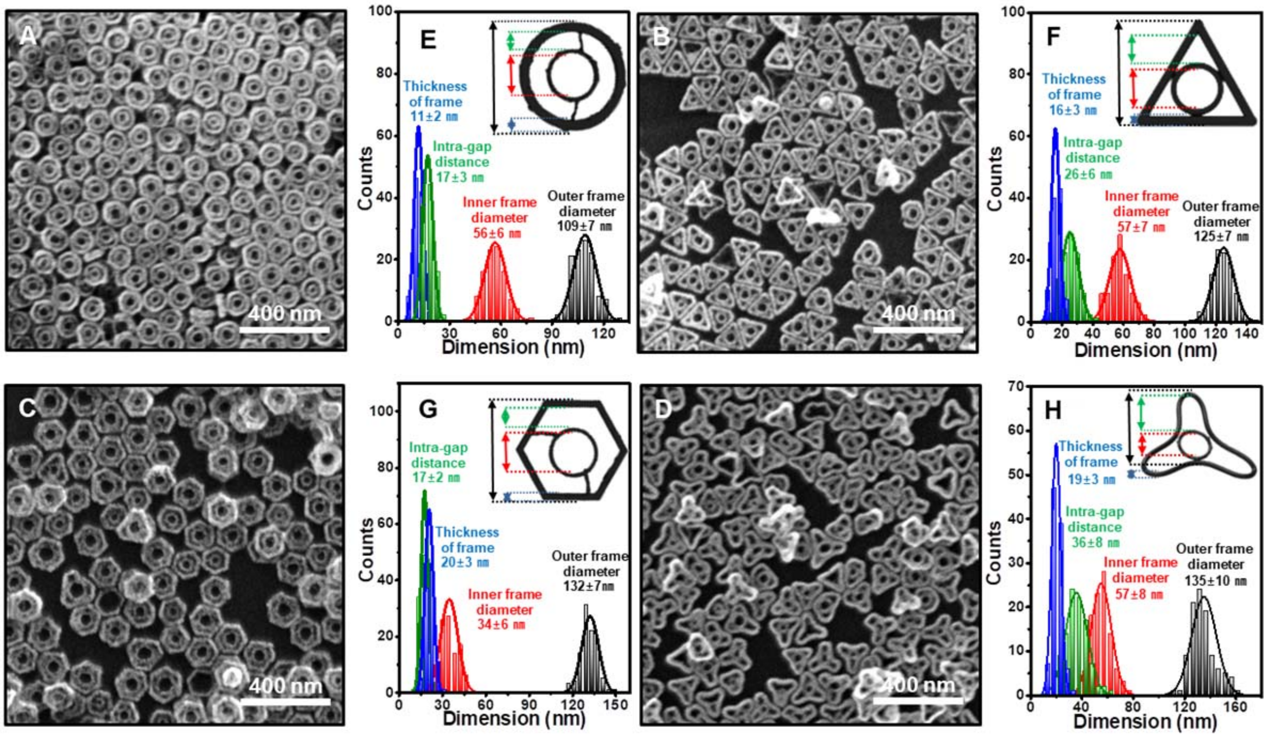

Supplementary Figures 6. Low-magnification SEM images of 2D PtAu double nanoframes of (A) disk, (B) triangle, (C) hexagon, and (D) tripod shape. Size distributions of PtAu double nanoframes of (E) disk, (F) triangle, (G) hexagon, and (H) tripod shapes, including outer frame diameter (black line), inner frame diameter (red line), intra-gap distance (green line) and frame thickness (blue line).

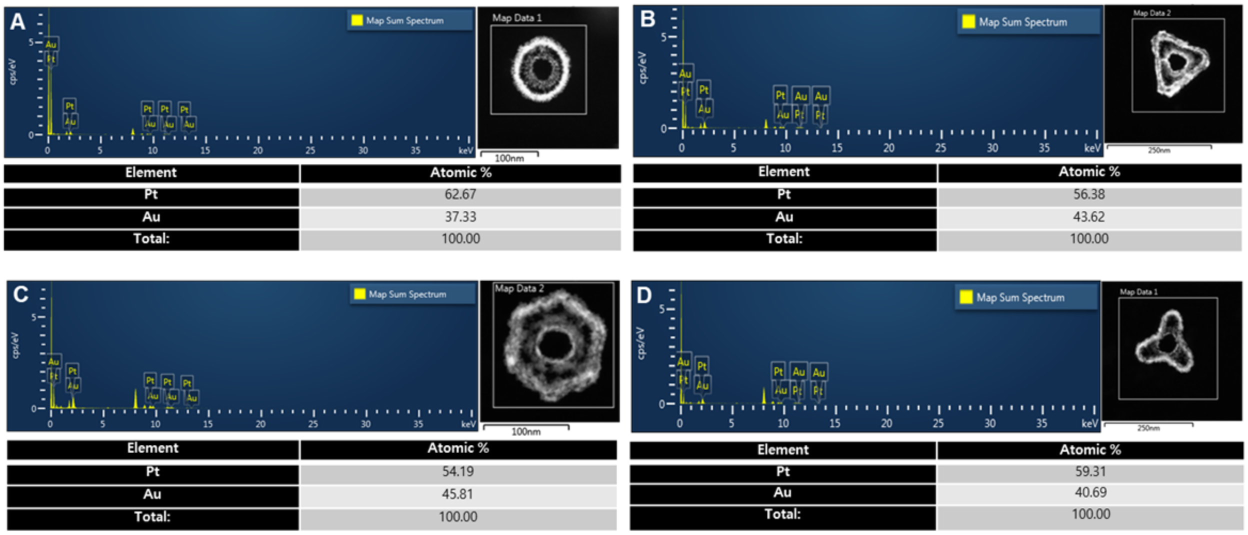

(A)

Element	Atomic %
Pt	62.67
Au	37.33
Total:	100.00

(B)

Element	Atomic %
Pt	56.38
Au	43.62
Total:	100.00

(C)

Element	Atomic %
Pt	54.19
Au	45.81
Total:	100.00

(D)

Element	Atomic %
Pt	59.31
Au	40.69
Total:	100.00

Supplementary Figures 7. EDS data indicating elemental proportions in 2D PtAu double nanoframes of (A) disk, (B) triangle, (C) hexagon, and (D) tripod shapes.

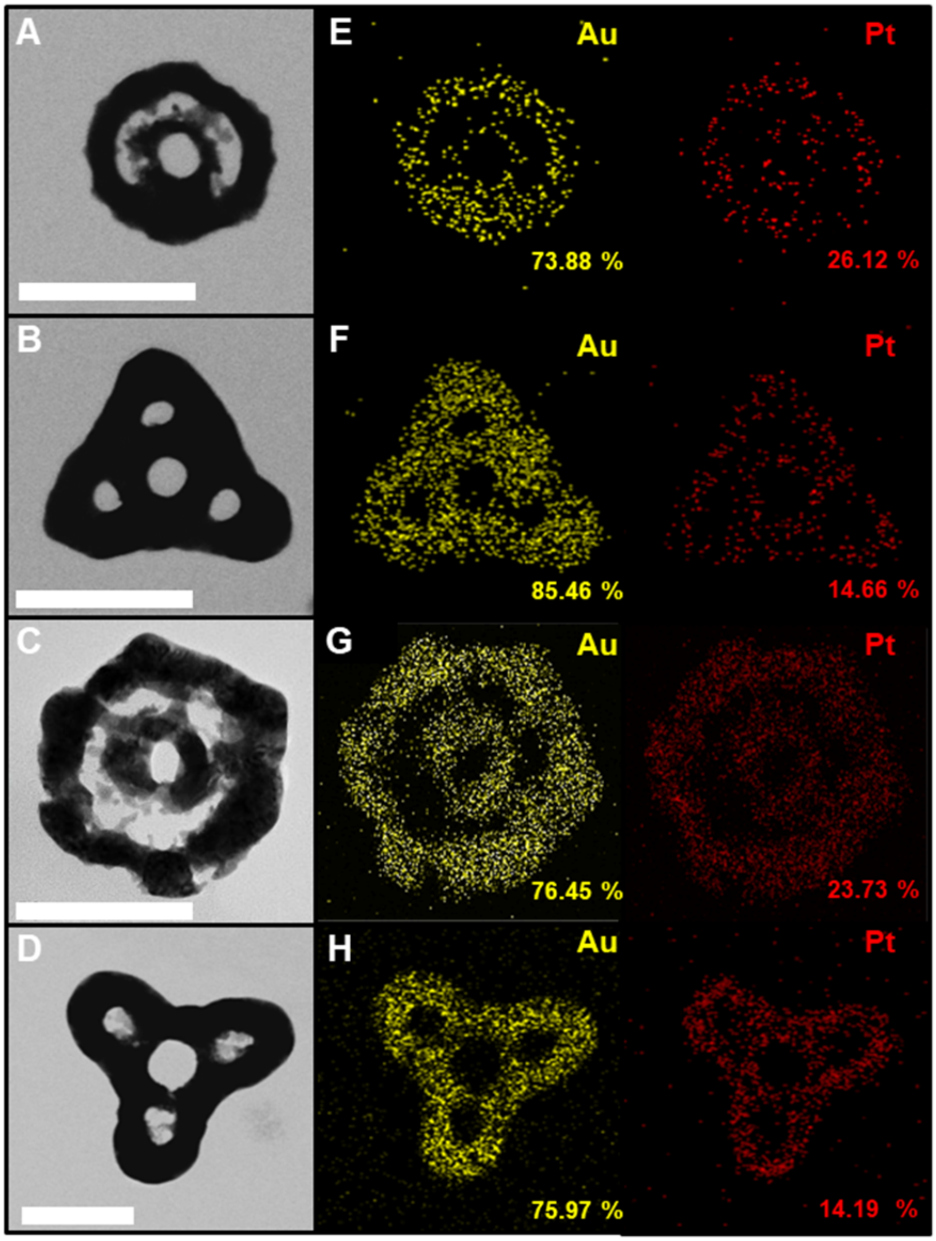

Supplementary Figures 8. TEM images and EDS image mappings of 2D Pt@Au double nanoframes of (A and E) disk, (B and F) triangle, (C and G) hexagon, and (D and H) tripod shapes (scale bars: 100 nm).

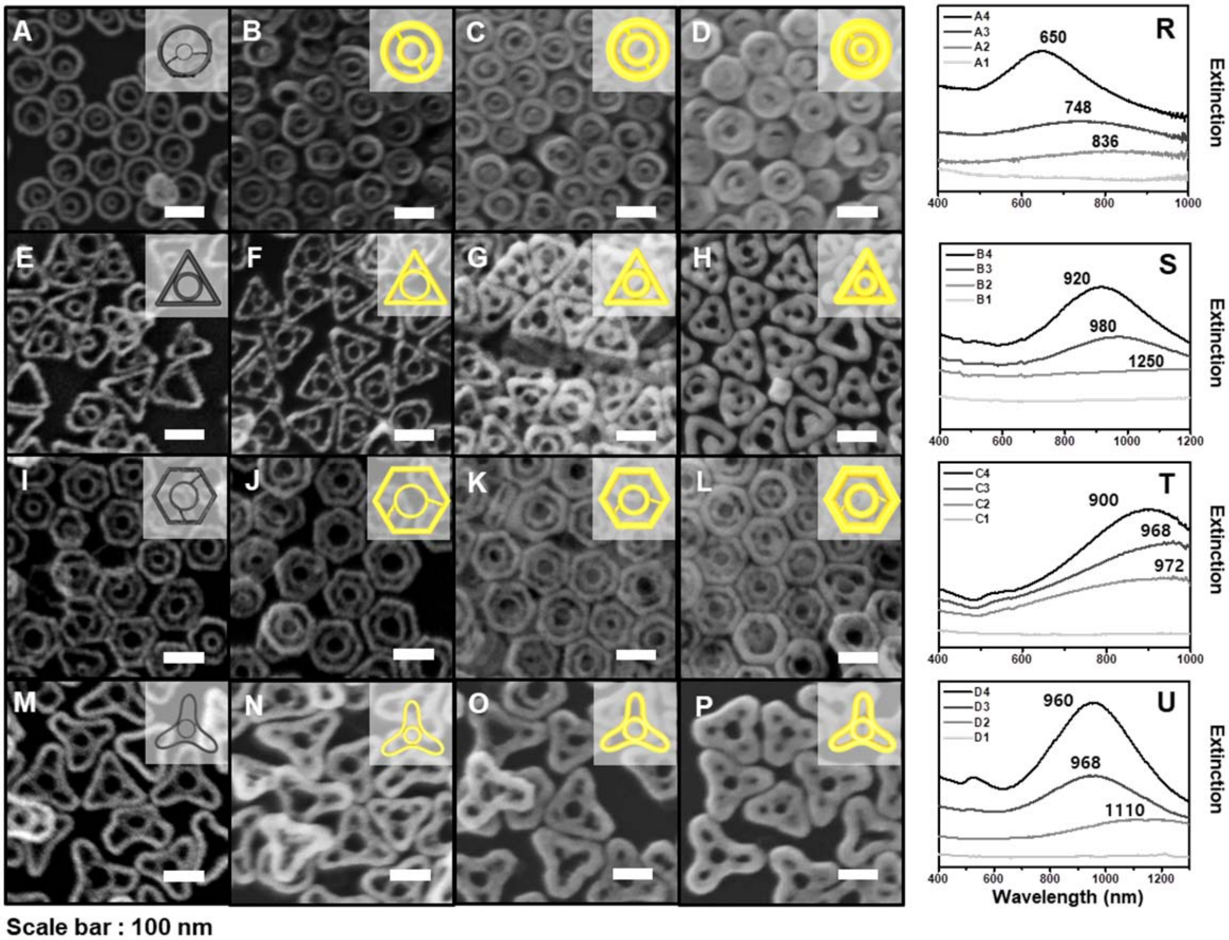

Supplementary Figures 9. FE-SEM images of PtAu double nanoframes of (A) disk, (E) triangle, (I) hexagon and (M) tripod shapes, and of Pt@Au double nanoframes of (B-D) disk, (F-H) triangle, (J-L) hexagon and (N-P) tripod shapes. Some of these have concentric growth mode depending on the concentration of $HAuCl_4$; 2 mM $HAuCl_4$ aqueous solution was added to the reaction solution containing the $AgNO_3$ ions in the volumes of (B-D and F-H) 100, 200 and 300 μL, (J-L) 100, 200 and 400 μL, and (N-P) 120, 360 and 480 μL. (R-U) UV-Vis-NIR spectra obtained from the samples shown in the SEM images, in order of increasing Au^{3+} ions (from pale gray to black).

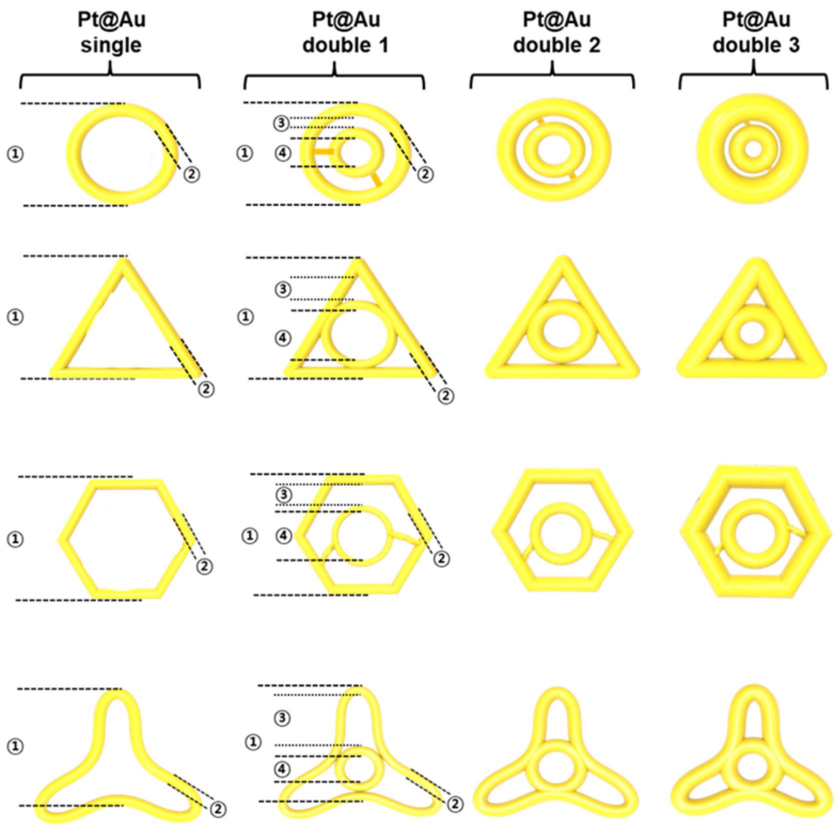

	Pt@Au single	Pt@Au double 1	Pt@Au double 2	Pt@Au double 3
①	103 nm	103 nm	110 nm	120 nm
②	15 nm	15 nm	20 nm	25 nm
③		14 nm	10 nm	5 nm
④		19 nm	15 nm	10 nm

	Pt@Au single	Pt@Au double 1	Pt@Au double 2	Pt@Au double 3
①	117 nm	117 nm	117 nm	125 nm
②	15 nm	15 nm	16 nm	23 nm
③		24 nm	17 nm	13 nm
④		27 nm	23 nm	16 nm

	Pt@Au single	Pt@Au double 1	Pt@Au double 2	Pt@Au double 3
①	130 nm	130 nm	132 nm	135 nm
②	16 nm	16 nm	13 nm	9 nm
③		20 nm	13 nm	9 nm
④		30 nm	26 nm	19 nm

	Pt@Au single	Pt@Au double 1	Pt@Au double 2	Pt@Au double 3
①	146 nm	146 nm	166 nm	180 nm
②	26 nm	26 nm	30 nm	36 nm
③		42 nm	30 nm	22 nm
④		32 nm	24 nm	16 nm

Supplementary Figures 10. Dimensions of Pt@Au single nanoframes and Pt@Au double nanoframes of different shapes.

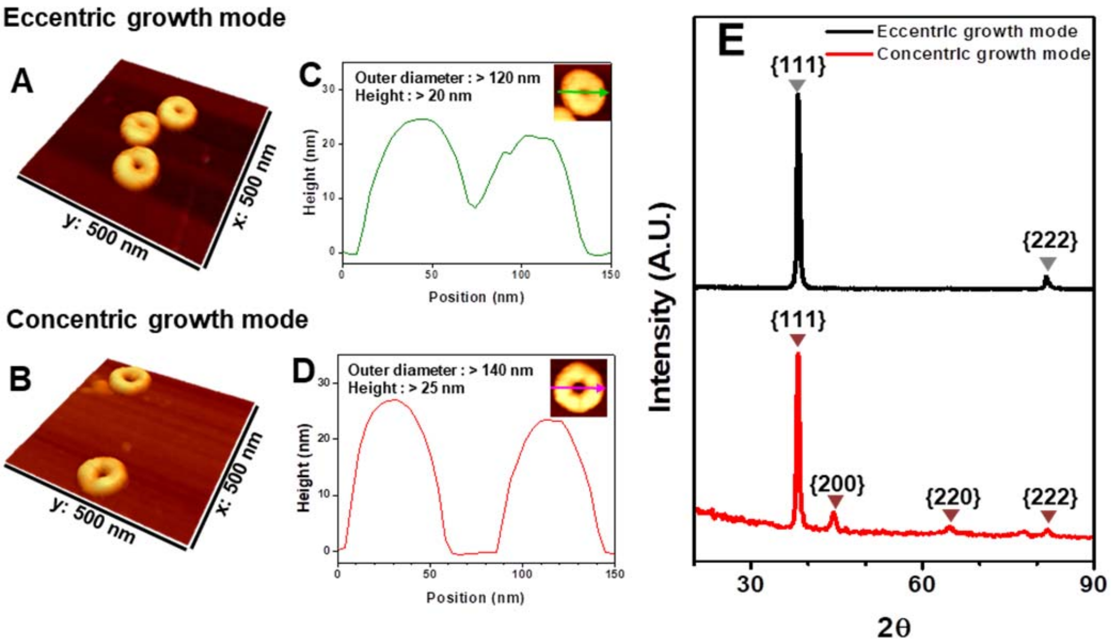

Supplementary Figures 11. Atomic force microscopy images of Au nanorings grown in (A) eccentric and (B) concentric growth mode. Atomic force microscopy height profiles of Au nanorings grown in (C) eccentric and (D) concentric growth mode along the red line in the right upper inset. (E) X-ray diffraction (XRD) pattern of Au nanorings grown in (black line) eccentric and (red line) concentric growth mode.
