# Supplementary material for: Two-dimensional nanoframes with dual rims
Source: Nat Commun. 2019 Dec 19;10:5789. doi: 10.1038/s41467-019-13738-6 (PMC6923375; doi:10.1038/s41467-019-13738-6)
Supplement: Supplementary file 2 — Description of Additional Supplementary Files [file 41467_2019_13738_MOESM2_ESM.pdf]

Description of Additional Supplementary Files

File Name: Supplementary Movie 1
Description: 3D STEM tomography movie of 2D PtAu double nanoframes of ring shape

File Name: Supplementary Movie 2
Description: 3D STEM tomography movie of 2D PtAu double nanoframes of triangle shape

File Name: Supplementary Movie 3
Description: 3D STEM tomography movie of 2D PtAu double nanoframes of hexagon shape.

File Name: Supplementary Movie 4
Description: 3D STEM tomography movie of 2D PtAu double nanoframes of tripod shape.
